# Rhino-orbital mucormycosis in COVID-19 patients—a new threat?

**DOI:** 10.1186/s43055-021-00535-9

**Published:** 2021-06-22

**Authors:** Sandeep Singh Awal, Som Subhro Biswas, Sampreet Kaur Awal

**Affiliations:** 1 Department of Radiology, Jeevandeep Diagnostics, Jamshedpur, India; 2 Department of Radiology, Radiance Teleradiology Services, Navi Mumbai, India; 3 Department of Microbiology, Guru Gobind Singh Medical College & Hospital, Faridkot, Punjab India

**Keywords:** COVID-19, Mucormycosis, Black turbinate sign, MRI, Case report

## Abstract

**Background:**

Coronavirus disease 2019 (COVID-19) is known to be associated with a myriad of viral, fungal, and bacterial co-infections. Rhino-orbital mucormycosis is a rare angio-invasive fungal infection which has shown a rising trend in the setting of COVID-19.

**Case presentation:**

We describe the imaging findings in 3 cases of rhino-orbital mucormycosis in patients with history of COVID-19. All cases had varying involvement of paranasal sinuses extending into the orbital compartment while case 3 had intracranial extension of infection.

**Conclusions:**

Rhino-orbital mucormycosis can have aggressive necrosis of the involved paranasal sinuses and orbits with or without cerebral extension. Hence, the correct diagnosis is imperative as prompt antifungal drugs and surgical debridement can significantly reduce mortality and morbidity.

## Background

The 2019 novel coronavirus disease (COVID-19) is an infectious viral disease caused by the severe acute respiratory syndrome coronavirus 2 (SARS-CoV-2) [1]. The ongoing COVID-19 outbreak has spread worldwide since the first human case was detected in the month of December 2019 [2, 3]. COVID-19 is known to cause respiratory symptoms primarily, ranging from mild to severe pneumonia [4]. However, it can be associated with a broad-spectrum of bacterial and fungal co-infections [5]. The current second wave of the COVID-19 pandemic in India has seen a rise in the rhino-orbital mucormycosis co-infections in COVID-19 patients [6].

Rhino-orbital mucormycosis is a rare invasive fungal infection that originates in the paranasal sinuses and may frequently extend into the orbits and cerebral parenchyma [7]. Uncontrolled diabetes mellitus and the use of corticosteroids for the treatment of respiratory symptoms are possible etiological factors [6, 7]. Mucormycosis can be life threatening as it has a high mortality rate of over 50% [8]. Early diagnosis, delineating the extent of the spread of infection, is necessary as medical and surgical intervention can reduce mortality and morbidity [7]. Hence, it is crucial for all radiology departments to be familiar with the imaging features of rhino-orbital mucormycosis.

We present the imaging findings in 3 cases of rhino-orbital mucormycosis co-infections in COVID-19 patients. Contrast-enhanced MRI (CE-MRI) protocol of the paranasal sinuses, brain, and orbits included axial T1, T2, T2 FLAIR, GRE, DWI, T2 FS, T1 FS post-contrast (3 mm thickness), sagittal T2, T1 FS post-contrast (3 mm thickness), coronal T2, and T1 FS post-contrast (3 mm thickness) sequences. High-resolution CT thorax (HRCT thorax) of case 1 was done using 32 slice multidetector CT machine using thin sections (1 mm slice thickness). Histopathological evaluation of nasal discharge of all 3 cases was done on potassium hydroxide (KOH) wet mount and further confirmed on culture using lactophenol cotton blue (LPCB) stain. Final diagnosis of mucormycosis was made based on clinical details, imaging findings, and histopathology.

## Case presentation

### Case 1

A 65-year-old diabetic female presented with respiratory distress, left orbital pain, left-sided ptosis, nasal congestion and discharge, and fever of short duration. Reverse transcriptase-polymerase chain reaction (RT-PCR) from nasopharyngeal swab for COVID-19 was positive.

**Fig. 1:**
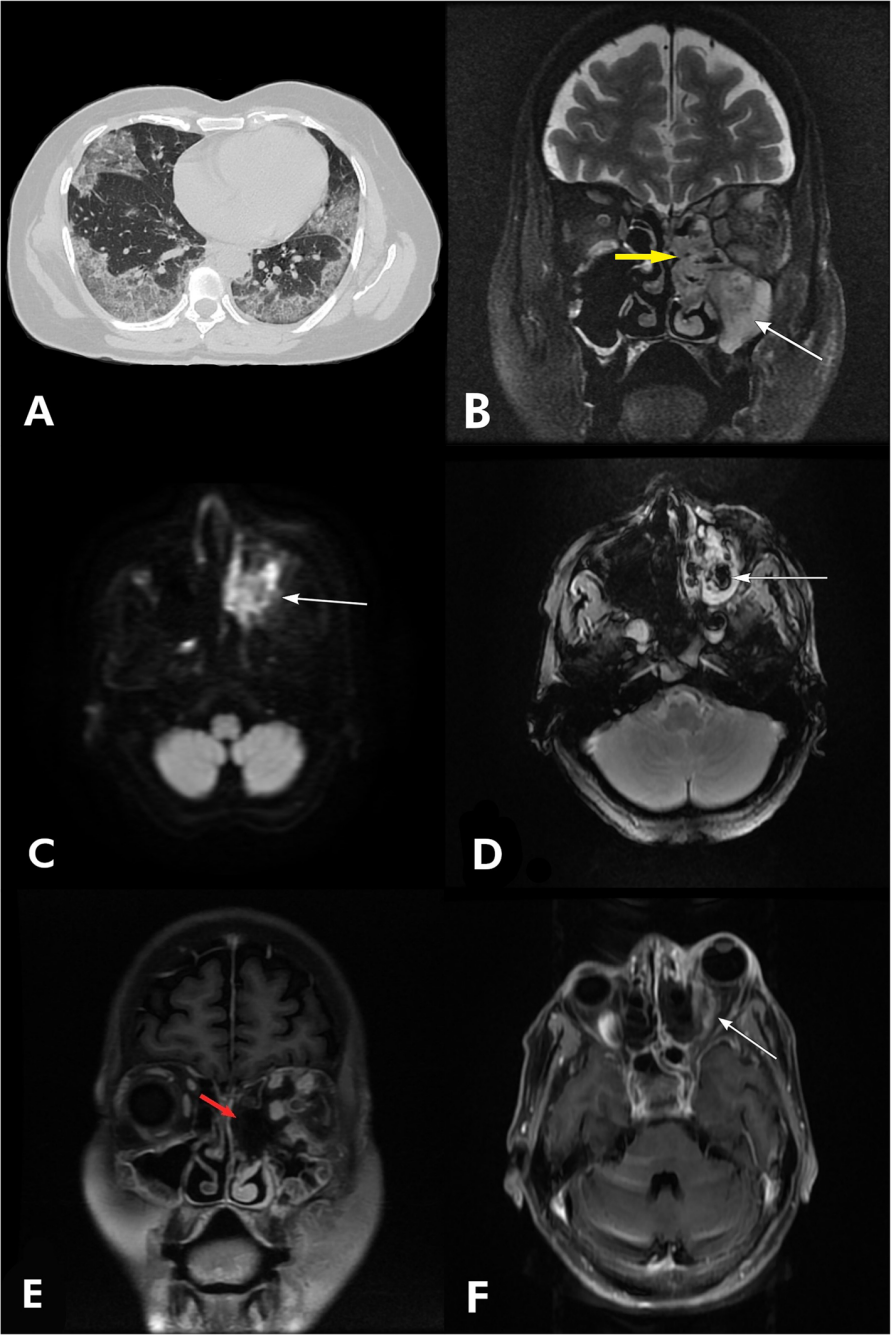
**a** Axial HRCT thorax image showing ground-glass opacities in subpleural regions of bilateral lung parenchyma with “crazy paving appearance.” **b** Coronal T2 FS image showing mucosal thickening and collection in the left maxillary sinus (white arrow), left ethmoidal sinus, and left middle nasal turbinate (yellow arrow) with inflamed extraocular muscles. **c** Axial DWI image showing restricted diffusion in the left maxillary sinus and left middle nasal turbinate. **d** Axial GRE image showing foci of blooming in the left maxillary sinus (white arrow). **e** Coronal T1 post-contrast image showing area of non-enhancing soft tissue in left middle nasal turbinate and within the left maxillary antrum (“black turbinate sign”). **f** Axial T1 post-contrast image showing enhancement and inflammation involving extraocular muscles of left orbit causing proptosis

HRCT thorax (Fig. 1a) revealed multiple peripheral ground glass opacities in posterior subpleural regions of bilateral lung parenchyma with interlobular septal thickening (crazy paving appearance) [9].

CE-MRI of the paranasal sinuses and orbits revealed mucosal thickening and collection in all paranasal sinuses, predominantly in left maxillary (Fig. 1b), sphenoidal, and ethmoidal sinuses. Restricted diffusion on DWI (Fig. 1c) and blooming on GRE (Fig. 1d) were seen involving left middle nasal turbinate and left maxillary sinus. Post-contrast T1-weighted images (Fig. 1e) showed enhancement in the involved structures with area of non-enhancing soft tissue in left middle nasal turbinate and within the left maxillary antrum (“black turbinate sign”) [10, 11]. Retroorbital fat, extraocular muscles of left orbit showed enhancement and inflammation on post-contrast T1 images with left sided proptosis (Fig. 1f).

**Fig. 2:**
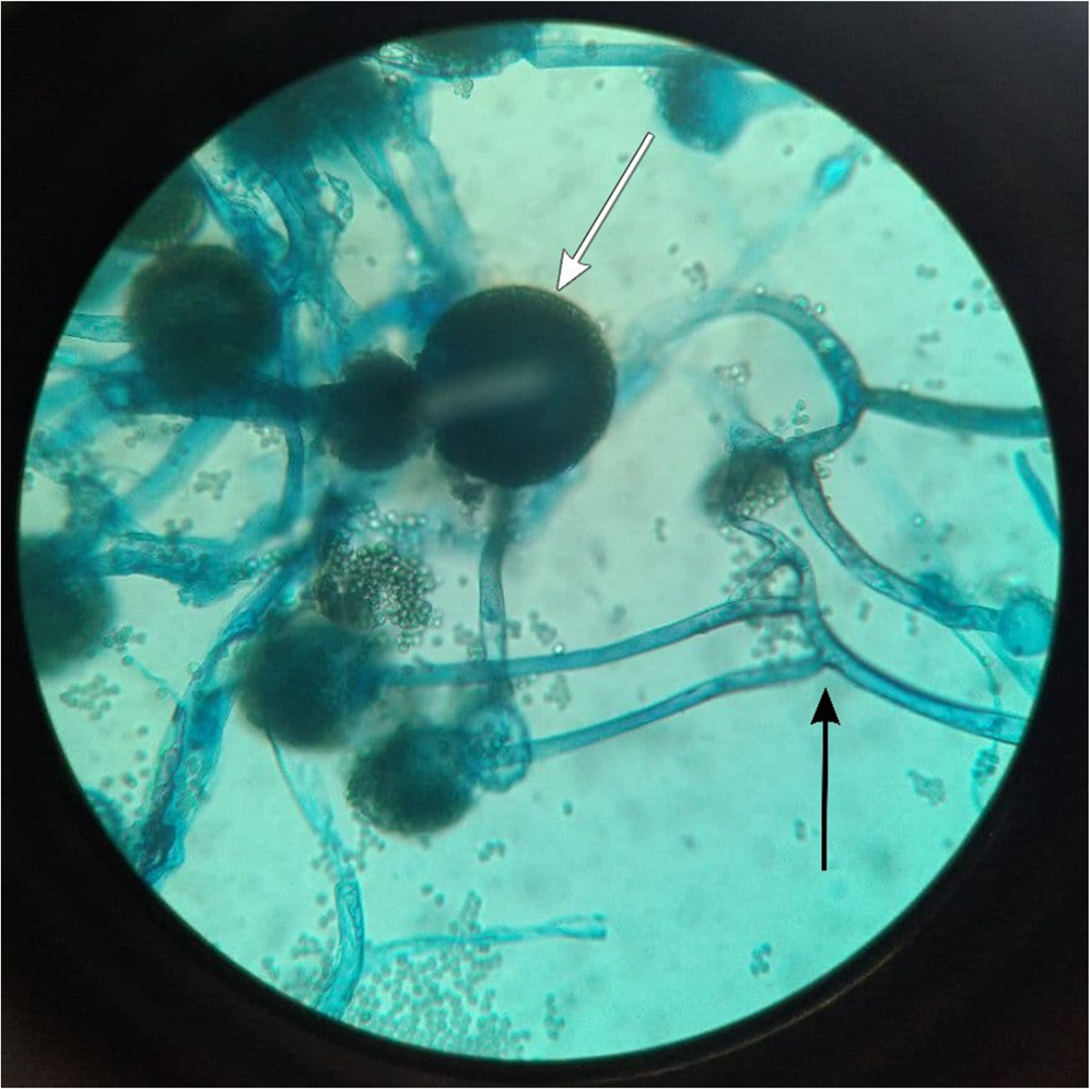
Lactophenol cotton blue (LPCB) stain after culture on Sabouraud dextrose agar (SDA) showing broad aseptate ribbon-like hyphae branching at right angles (black arrow) with sporangium (white arrow)

Histopathological evaluation (HPE) of the nasal discharge revealed broad aseptate ribbon-like fungal hyphae on KOH wet mount [12]. Lactophenol cotton blue (LPCB) stain after 72 h of culture on Sabouraud dextrose agar (SDA) revealed broad aseptate ribbon-like hyphae branching at right angles with sporangium (Fig. 2).

### Case 2

A 45-year-old female presented with right hemifacial pain and right orbital swelling for 5 days. Patient had past history of severe COVID-19 pneumonia for which she was hospitalized 3 weeks ago. She was treated with remdesivir, oxygen support, and intravenous methylprednisolone.

**Fig. 3:**
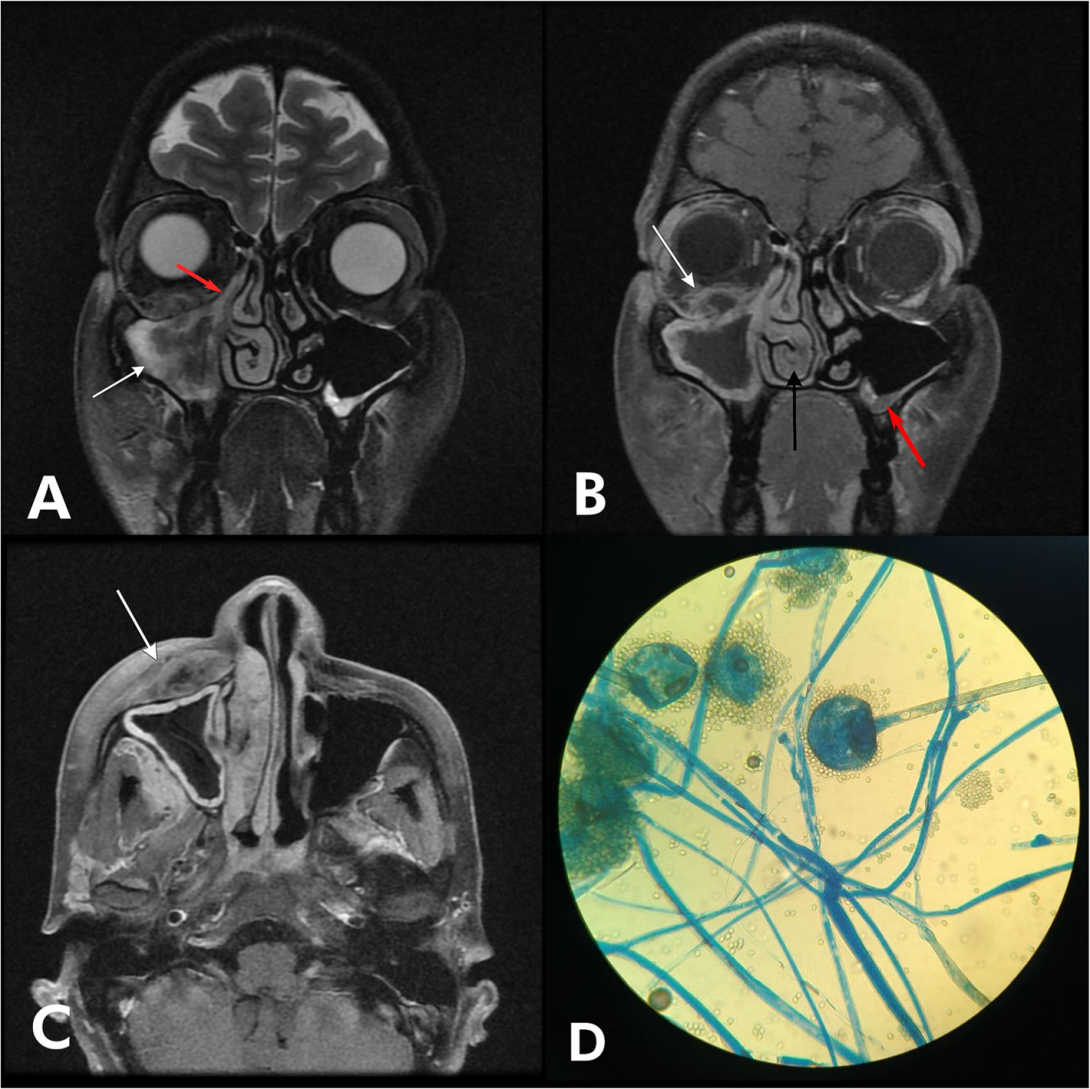
**a** Coronal T2 image showing mucosal thickening in right maxillary sinus (white arrow) causing blockage of the right osteomeatal unit (red arrow). **b** Coronal T1 FS post-contrast image showing extension of inflammation in right inferior orbit (white arrow) with post-contrast peripheral enhancement, right inferior turbinate hypertrophy (black arrow), and left maxillary wall thickening (white arrow). **c** Axial T1 FS post-contrast image showing right pre-maxillary soft tissue swelling with enhancement (white arrow). **d** Lactophenol cotton blue (LPCB) stain showing broad ribbon-like fungal hyphae with sporangium

CE-MRI of the paranasal sinuses and orbits revealed mucosal thickening and collection in the right maxillary sinus causing blockage of right osteomeatal unit (Fig. 3a). Extension of inflammation with heterogenous post-contrast peripheral enhancement was seen in the right inferior orbital wall (Fig. 3b). Hypertrophied right middle and inferior nasal turbinates. Soft tissue swelling involving the right pre-maxillary soft tissue was seen (Fig. 3c). Mucosal thickening also noted in the ethmoidal and left maxillary sinus (Fig. 3b). KOH mount of nasal discharge revealed broad aseptate fungal hyphae, later confirmed on culture showing broad ribbon-like hyphae with sporangium (Fig. 3d).

### Case 3

A 36-year-old male with recent history of COVID-19 pneumonia presented with persisting left hemifacial pain and rhinorrhea.

**Fig. 4:**
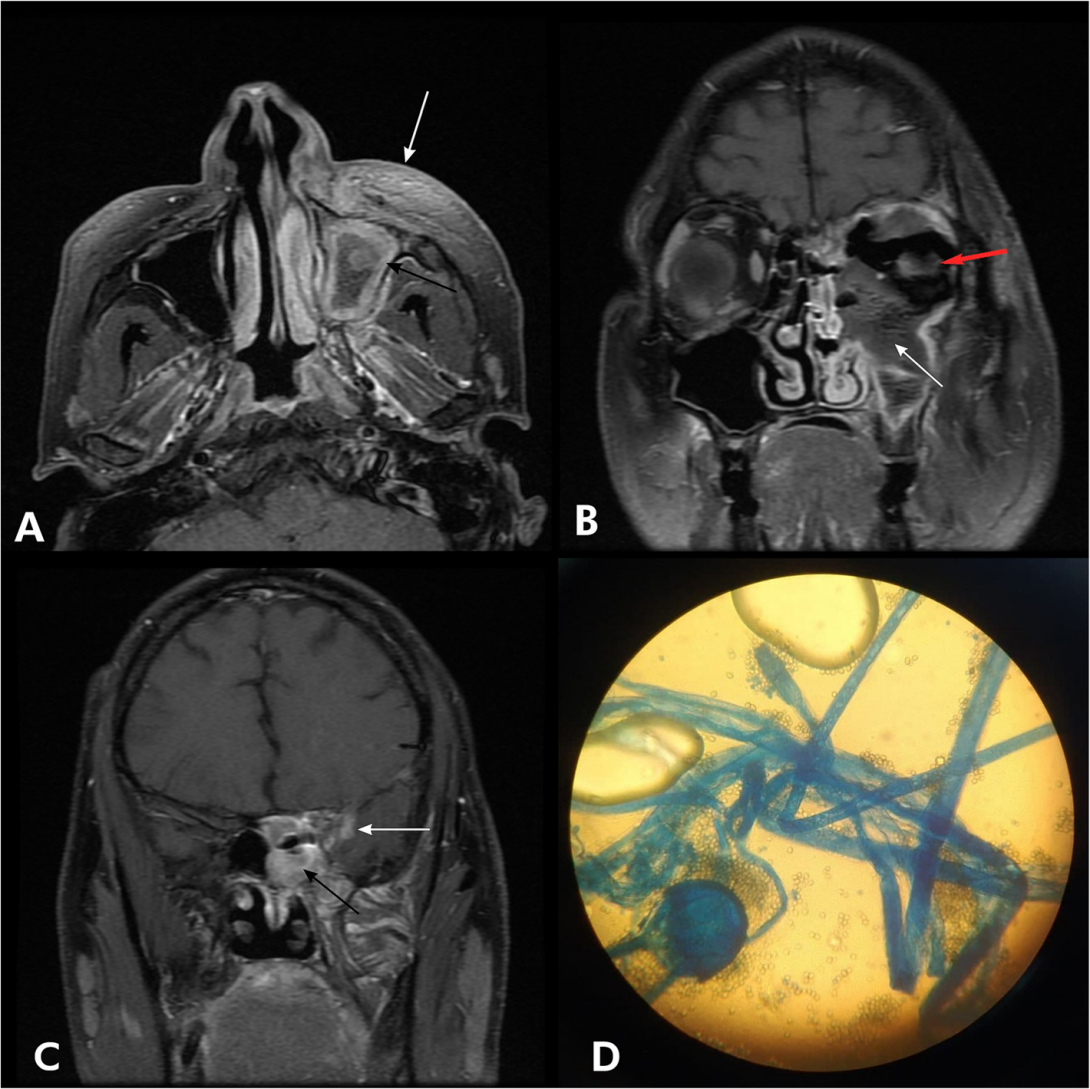
**a** Axial T1 post-contrast image showing mucosal thickening and collection involving left maxillary sinus (black arrow) with pre-maxillary soft tissue swelling (white arrow). **b** Axial T1 post-contrast image showing inferior orbital wall with extension of soft tissue component into left inferomedial orbit (white arrow) and left phthisis bulbi (red arrow). **c** Coronal T1 post-contrast image showing left sphenoid sinusitis (black arrow) with an area of enhancement involving left medial temporal lobe (white arrow). **d** Lactophenol cotton blue (LPCB) stain showing broad aseptate ribbon-like hyphae with sporangium

CE-MRI of the paranasal sinuses, brain, and orbits revealed mucosal thickening and collection involving frontal, ethmoidal, sphenoidal, and left maxillary sinuses (Fig. 4a). Left pre-maxillary soft tissue swelling was seen. Bony defects involving inferior orbital wall was seen with extension of soft tissue component into left inferomedial orbit (Fig. 4b). Small, shrunken left eye globe was seen suggestive of phthisis bulbi [13]. Enhancement involving left infratemporal fossa region and left medial temporal lobe was noted, suggestive of intracranial extension (Fig. 4c).

KOH wet mount revealed broad non septate fungal hyphae. Broad aseptate ribbon-like hyphae with sporangium is seen on the LPCB stain after 72 h of culture in SDA agar (Fig. 4d).

## Discussion

A myriad of co-infections of viral, fungal, and bacterial etiology and associated complications have been encountered in patients of COVID-19 [14, 15]. During the current second wave of the COVID-19 pandemic in India, an increase in fungal infections, predominantly rhino-orbital mucormycosis, has been documented [6].

Mucormycosis is a rare, opportunistic fungal infection which causes angio-invasive disease leading to aggressive necrosis and infarction of the involved tissues. Rhino-orbital mucormycosis involves the paranasal sinuses and orbits and may extend into the cerebral parenchyma [7, 15]. Underlying predisposing factors include uncontrolled diabetes mellitus, immunocompromised status, systemic use of corticosteroids, pre-existing respiratory pathology, cancer, and stem cell transplant [5, 7, 15]. Among these, diabetes is one of the most common etiological factors [16].

MRI is a valuable modality that can be used to diagnose mucormycosis infections involving sino-nasal region, orbits, and possible intracranial extension [17]. The multiplanar capabilities of MRI with its superior soft tissue depiction are helpful in delineating the anatomical extent of disease as well as its complications [17, 18].

Imaging findings of mucormycosis include mucosal thickening and/or opacification of the involved paranasal sinuses. Majority of the lesions appear hypointense on T1-weighted images and variable to hyperintense on T2-weighted images [17]. Low signal intensity of fungal elements on T2-weighted images along with restricted diffusion on DWI may be seen [11]. Hypertrophy of nasal turbinates with nasal secretions is seen with nasal involvement [18]. Post-contrast enhancement can be seen in the thickened mucosa and involved tissues. However, areas of non-enhancing soft tissue may be seen within the affected turbinates and/or paranasal sinuses, known as the “black turbinate sign” [10, 11]. This sign may help in the early detection of nasal mucormycosis [11].

Extrasinus extension into orbital compartment, face is commonly encountered and may further extend into the infratemporal fossa, cavernous sinus, skull base, and intracranial compartment [18]. Post-treatment follow-up MRI may be needed in some cases.

## Conclusions

Imaging plays a key role in the early identification of rhino-orbital mucormycosis and delineating the extent of infection. Prompt diagnosis and treatment of rhino-orbital mucormycosis is the “sine qua non” as antifungal drugs and surgical debridement can successfully control the infection and thus reduce the high mortality and morbidity associated with mucormycosis. Due to the changing trends in the COVID-19 pandemic, it is an absolute necessity for all radiologists to be well aware of the imaging features of rhino-orbital mucormycosis and its possible complications.

## Data Availability

The data and materials supporting the findings of this study are available on request from the corresponding author.

## Abbreviations

COVID-19: Coronavirus disease 2019
SARS-CoV-2: Severe acute respiratory syndrome coronavirus 2
RI: Magnetic resonance imaging
CE-MRI: Contrast-enhanced MRI
FLAIR: Fluid-attenuated inversion recovery
GRE: Gradient recalled echo
DWI: Diffusion-weighted imaging
FS: Fat saturated
HRCT: High resolution computed tomography
KOH: Potassium hydroxide
RT-PCR: Reverse transcriptase-polymerase chain reaction
HPE: Histopathological evaluation
LPCB: Lactophenol cotton blue
SDA: Sabouraud dextrose agar
